# The 30-kDa and 38-kDa antigens from *Mycobacterium tuberculosis* induce partial maturation of human dendritic cells shifting CD4^+^ T cell responses towards IL-4 production

**DOI:** 10.1186/1471-2172-14-48

**Published:** 2013-10-03

**Authors:** Marion Heuer, Anna-Sophie Behlich, Ji-Sook Lee, Eliana Ribechini, Eun-Kyeong Jo, Manfred B Lutz

**Affiliations:** 1 Institute of Virology and Immunobiology, University of Würzburg, Würzburg, Germany; 2 Department of Microbiology, College of Medicine, Konyang University, Daejeon 302-718, South Korea

**Keywords:** Dendritic cells, *Mycobacterium tuberculosis*, T helper cell responses

## Abstract

**Background:**

*Mycobacterium tuberculosis* (Mtb) infections are still a major cause of death among all infectious diseases. Although 99% of individuals infected with Mtb develop a CD4^+^ Th1 and CD8^+^ T cell mediated immunity as measured by tuberculin skin test, this results only in partial protection and Mtb vaccines are not effective. Deviation of immune responses by pathogens towards a Th2 profile is a common mechanism of immune evasion, typically leading to the persistence of the microbes.

**Results:**

Here we tested the stimulatory capacity of selective Mtb antigens on human monocyte-derived dendritic cell (DC) maturation and cytokine production. DC maturation markers CD80, CD86 and CD83 were readily upregulated by H37Ra- and H37Rv-associated antigens, the 30-kDa (from Ag85 B complex) and 38-KDa Mtb antigens only partially induced these markers. All Mtb antigens induced variable levels of IL-6 and low levels of IL-10, there was no release of IL-12p70 detectable. Substantial IL-12p40 production was restricted to LPS or H37Ra and H37Rv preparations. Although the proliferation levels of primary T cell responses were comparable using all the differentially stimulated DC, the 30-kDa and 38-kDa antigens showed a bias towards IL-4 secretion of polarized CD4^+^ T cells after secondary stimulation as compared to H37Ra and H37Rv preparations.

**Conclusion:**

Together our data indicate that 30-kDa and 38-kDa Mtb antigens induced only partial DC maturation shifting immune responses towards a Th2 profile.

## Background

From the people infected worldwide with Mtb only about 5-10% develope disease, while the majority can control the disease through NK cell and Th1 immune responses characterized by the release of IFN-γ and TNF leading to the cytotoxic activity of CD8^+^ T cells and macrophages. Susceptibility to Mtb is considered to result from a suboptimal immune response and therefore a vaccine against Mtb would improve this status. However, no vaccine based on Mtb is currently available. BCG as an attenuated strain of *M. bovis* has been used as a vaccine against Mtb but without achieving a reliable protection [1]. Thus, alternative vaccination strategies are urgently needed [2].

Currently modified BCG vaccines are the most common tested in clinical trials but also few selective Mtb antigens have been tested for their capacity to stimulate immune responses in order to use them as a vaccine [3]. A successful vaccine should induce strong CD8 and Th1 memory responses and at the same time avoid the induction of immune tolerance mechanisms. Immune deviation towards Th2 responses is a hallmark of many infections leading to microbial persistence [4]. Thus, we wanted to investigate whether immune deviation could be detected by selective Mtb components. We studied these factors not as antigens presented on MHC molecules but as factors to induce DC maturation. The quality of DC maturation was then assessed as well as the DC-mediated immune responses of CD4^+^ T cells.

Initially, all types of DC maturation were believed to induce DC immunogenicity. By establishing a semi-mature stage of DC maturation we could demonstrate that matured DC nevertheless could act tolerogenic. TNF treatment of murine bone marrow-derived DC led to their partial maturation and after i.v. injection induced protective IL-10 producing T cells (Tr1) in the model of experimental autoimmune encephalomyelitis (EAE) [5]. This effect was antigen-specific and not dependent on bystander proteins [6]. Cytokine analysis revealed that also IL-4 and IL-13 produced from CD4^+^ T and NKT cells contributed to the protection, indicating a Th2 cell involvement [7]. Interestingly, although injections of TNF/DC induced a mixed Tr1/Th2 response when injected alone, antigen-specific pre-treatment of mice with TNF/DC did not boost subsequent Th2 cell responses such as *Leishamania major* infection of BALB/c mice [8] or allergic asthma [9]. This effect of tolerogenic mature DC is not restricted to the murine system. Others showed that TNF/PGE_2_-maturation of human monocyte-derived DC was required to perform cross-tolerance [10]. Thus, DC maturation must not necessarily indicate the induction of protective immunity.

Membrane and secreted molecules but also whole protein extracts of Mtb represent promising candidates in Mtb vaccine development. The Ra and Rv strains have been studied extensively and recent gene array analysis indicates that the Rv strain is by far better in promoting Th1 responses [11]. In this study, culture filtrate proteins (CFPs) isolated from Mtb H37Rv and H37Ra strains [12] were compared to find out whether attenuation versus virulence would induce differences in CD4^+^ T cell polarization. The 38-kDa protein represents an immunodominant phosphate-binding protein that was originally identified in pulmonary tuberculosis and a model antigen to screen for Mtb infections [13]. In mycobacterial culture fluid the antigen 85 complex B (Ag85B) is a major secretory component of Mtb that is also considered as a candidate for a vaccine due to its protection in animal experiments. The already commercially available 30-kDa protein is part of the Ag85B complex and when expressed in BCG shows more potent protection against Mtb [13]. Thus CFP preparations as well as these two immunodominant secretory proteins 30-kDa and 38-kDa antigens derived from the virulent strain Mtb H37Rv [14,15] may represent candidates for vaccine development. Since effective anti-mycobacterial immune responses are of the Th1 and not Th2 type, we developed a human CD4+ T cell polarization system to test these antigens for their potential to shift immune responses towards Th2 as a sign of immune evasion.

Here we addressed the questions whether the 30-kDa, 38-kDa or CFP preparations from H37Rv and H37Ra were able to mount DC maturation *in vitro* and to instruct the DC for a subsequent polarization of CD4^+^ T-helper (Th) cell responses. To enable the polarization of a substantial frequency of CD4^+^ T cells, we used the superantigen SEB. The results indicate a partial activation/maturation of DC as detected by surface markers and cytokine production. The 30-kDa and 38-kDa Mtb antigens appeared weaker DC stimulators than the Ra and Rv preparations. Primary T cell stimulations by these DC revealed similar proliferation rates. However, the secondary stimulation leading to polarized CD4+ T cell responses showed some differences. While robust IFN-γ production could be observed by all Mtb preparations, it was of note that the 30 kDa and 38 kDa antigens also maintained IL-4 production as a sign of immune deviation.

## Results and discussion

### The 30-kDa and 38-kDa Mtb antigens induce only a mild upregulation of DC maturation markers and cytokine production

**Figure 1:**
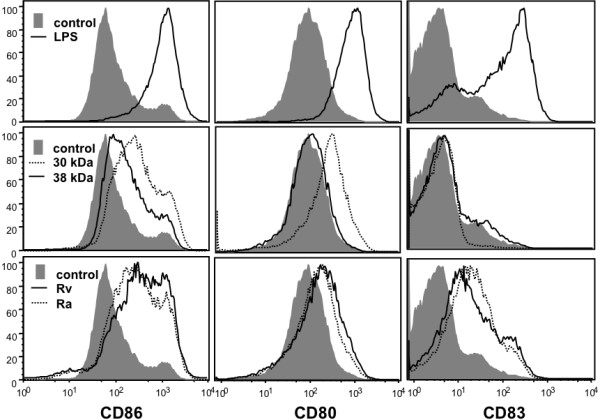
**DCs show only a partial maturation on 30-kDa and 38-kDa Mtb antigen stimulation.** DCs were generated from peripheral blood monocytes of healthy donors and at day 5 of culture stimulated with 10 μg of the indicated Mtb antigens or LPS or left untreated in buffer as a control for 24 h. Then cells were stained at the cell surface for the indicated markers (dotted or straight lines). Gray histograms show the markers on immature DC (control). The experiment is representative of 5 independent experiments.

Various mycobacterial components induce maturation of DCs but mostly Mtb surface products have been investigated [16]. The immunogenicity of the different Mtb preparations was tested by their incubation with human monocyte-derived DC *in vitro*. After 24 h of stimulation positive control DC that were stimulated with LPS, strongly upregulated the key surface markers CD80, CD83 and CD86 (Figure 1). The Mtb preparations H37Rv and H37Ra showed a low stimulatory capacity and the 30-kDa and 38-kDa preparations had only a weak or even no effect on these markers (Figure 1). In comparison to the strong maturation by Mtb surface products that was similar to LPS [16], only a mild upregulation of typical maturation markers was observed here.

**Figure 2:**
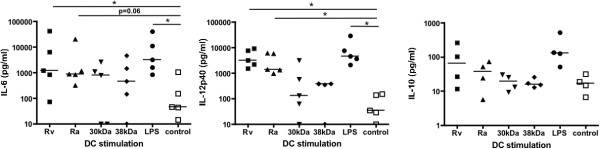
**Mtb antigens lead to moderate cytokine production of DCs.** DCs were generated from peripheral blood monocytes of healthy donors and at day 5 of culture stimulated with 10 μg of the indicated Mtb antigens or LPS or left untreated in buffer as a control for 24 h. The indicated cytokines were detected from the cell supernatants. The two experiments shown were performed from two different donors and show high inter-individual variations. They are representative of 5 independent experiments. Error bars represent the SEM of duplicate samples from the ELISA.

To further test the DC maturation capacity of Mtb antigens, supernatants of the stimulated cells were analysed for their cytokine content. Surprisingly, only the LPS control revealed measurable amounts of IL-12p70 but not the mycobacterial antigens (not shown). While IL-6 release by DCs was variable and highly donor-dependent, IL-10 production could be observed at low amounts by LPS, H37Rv and H37Ra (Figure 2). Consistently IL-12p40 was induced by LPS, H37Rv and H37Ra but only at low concentrations of below 1 ng/ml by the 30-kDa and 38-kDa antigens (Figure 2).

These data indicate that especially the 30-kDa and 38-kDa preparations induce only a partial DC maturation that may lead to a tolerogenic or immune deviatory CD4^+^ Th2 cell response as observed in murine models [5,8]. In fact, more recent gene profiling experiments indicate that murine DCs matured only partially e.g. with TNF or *Trypanosoma brucei* antigens show a common signature of only 24 regulated inflammatory genes identified for Th2-inducing DCs, whereas the same gene set as well as additional other genes induced by bacterial LPS result in full DC maturation and Th1 induction [9]. Interstingly, after restimulation of peripheral blood cells from patients with recurrent tuberculosis with the 30-kDa antigen we found depressed levels of IL-12 previously [17]. In the following experiments we addressed the question whether such quantitative differences in DC maturation may affect T cell priming capacity or the polarization into Th1/Th2 responses. From our murine data we hypothesized that also partial human DC maturation as observed for the 30-kDa and 38-kDa preparations may lead to minor differences in the T cell proliferation but significantly alter Th1/Th2 cytokine profiles.

### Similar allogeneic and SEB-dependent T cell priming capacity by the differentially Mtb-matured DC

**Figure 3:**
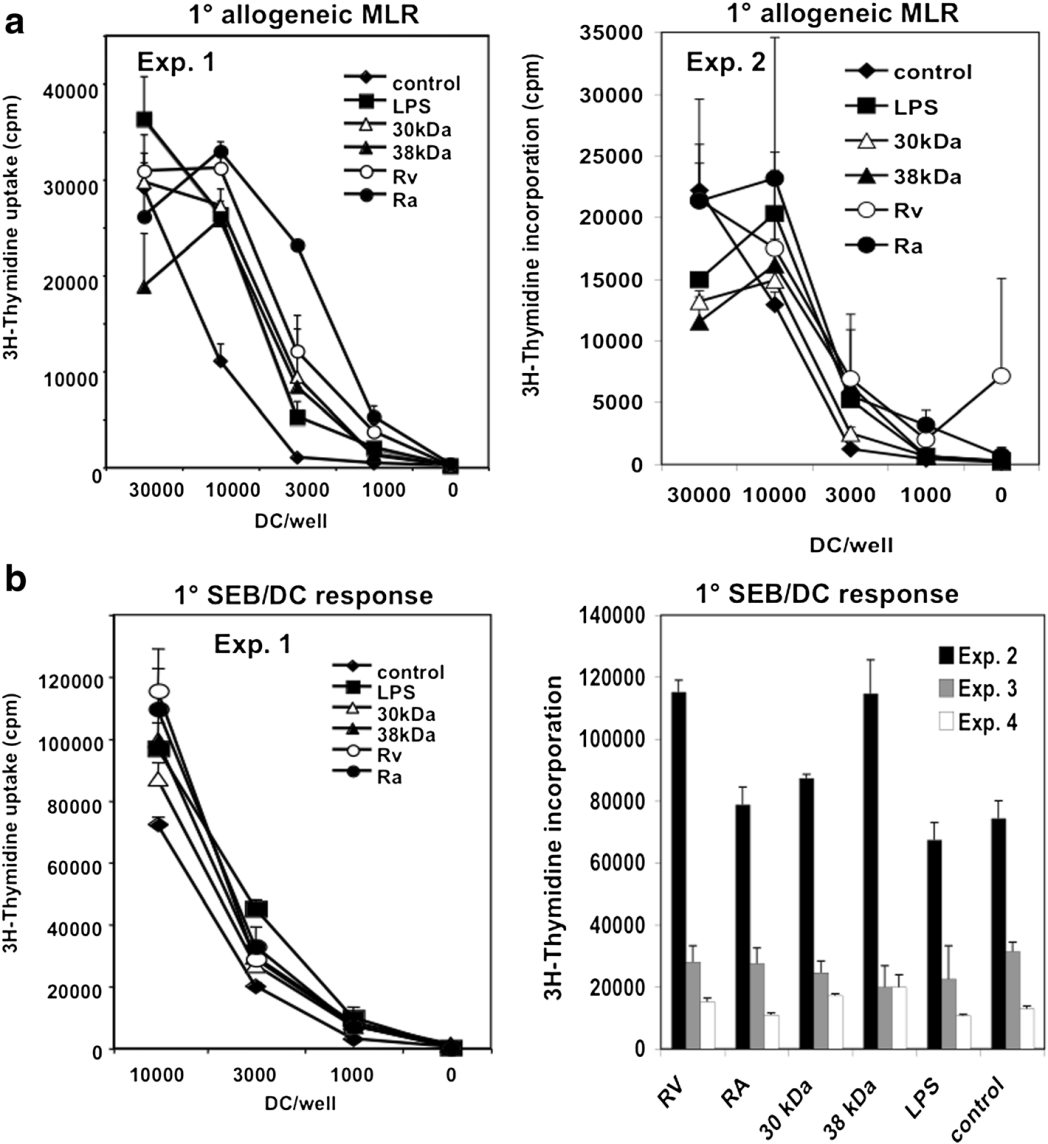
**Mtb-matured DCs show similar allogeneic and SEB-specific T cell priming capacities.** DC were stimulated at day 5 for 24 h with the indicated reagents and then used to stimulate allogeneic T cells **(a)** or autologous T cells in the presence of SEB **(b)**. After 3 days proliferation was assessed by [^3^H]-thymidine incorporation. **a**. The two experiments shown are representative of 3 independent experiments. Error bars represent the SEM of triplicate samples from one proliferation assay. **b**. The data of 4 experiments are shown. For experiment 1 the error bars represent the SEM of triplicate samples from one proliferation assay. For the experiments 2–4 data for the highest/optimal proliferation values of each experiment are shown. Error bars represent the SEM of triplicate samples of one proliferation assay. Note high variations between the different donors.

We then analysed whether the differential DC maturation conditions would result in diverging T cell priming capacities. When the differentially matured DCs were used to prime allogeneic T cells *in vitro* all stimuli appeared remarkably similar to the LPS control (Figure 3a). Similar proliferation rates were also obtained by stimulating syngeneic T cells with the differentially matured DCs and the superantigen SEB (Figure 3b). Thus, despite strong differences on DC maturation markers and cytokine profiles, the capacities to stimulate primary T cells did not differ substantially between the matured DC. The discrepancies between diverging surface marker expressions by DCs but similar T cell proliferation rates may indicate that not the quantity of the T cell response but the quality may have changed. Former observations in the murine system showed that DCs that were stimulated with *Trypanosoma* antigens induced very similar proliferation rates of T cells after injection *in vivo*[9]. However, the qualities of T helper cell polarization differed markedly. Therefore we set up a CD4^+^ T cell proliferation assay where polarization of T cells could be tested.

### The 30-kDa and 38-kDa Mtb antigens maintain Th2 cell responses

**Figure 4:**
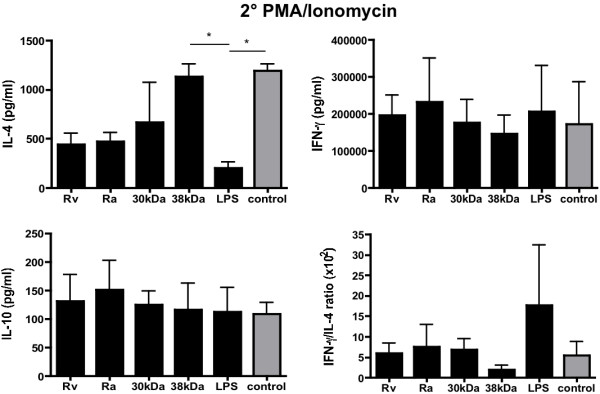
**DCs stimulated with the 30-kDa and 38-kDa Mtb antigens lead to a Th2 shift *in vitro*.** DCs were stimulated with the different Mtb antigens or LPS or left untreated and simultaneously loaded with the superantigen SEB. After washing the DCs were co-cultured with autologous T cells for 7 days. Then T cells were restimulated for 3 days by PMA/Ionomycin to induce cytokine release. Cytokines were measured by ELISA. Error bars represent the pooled mean data from 4 independent experiments. The IFN-γ/IL-4 ratio was calculated from 4 experiments and the pooled ratios are shown. Statistical significance was tested using one-way ANOVA test with Bonferroni’s post test. * = p < 0.05.

Previously we found that a quantitatively weaker T cell stimulation by DC may result in the same T cell proliferation rates but different polarizations into CD4^+^ T helper (Th) cell subsets [18]. Since the frequency of naive antigen-specific T cells from PBMCs is too low to be followed by any MHC II-restricted peptide stimulation *in vitro*, we used a superantigen system where higher naive antigen-specific frequencies of T cells can be activated during T cell priming [19]. In this system we explored the Th1 or Th2 subset polarization of the Mtb antigen-matured DC. Primary SEB-stimulated T cells were restimulated after 7 days with PMA/Ionomycin and their supernatants tested for cytokine release. All groups of stimulated T cells released substantial amounts of IFN-γ and IL-10 with no significant differences (Figure 4). However, T cells that were stimulated by 30-kDa- or 38-kDa-matured DC maintained a high IL-4 release as compared to immature (SEB-treated) DC, although the 30-kDa protein showed only as a trend (Figure 4). When the IFN-γ/IL-4 ratio was calculated the 38-kDa antigen showed the strongest IL-4 bias although no significance was achieved by this calculation (Figure 4). These data indicate that DCs matured with the 38-kDa antigen and as a trend also with the 30-kDa antigen, allow SEB-specific T cell responses biased towards a Th2 profile, similar to immature (SEB-treated) DCs.

Although immune responses tested at the level of DCs against intracellular Mtb and many of their antigens are dominated by IFN-γ producing CD8^+^ killer T cell and Th1 responses [20], also Th2 responses have been observed after infection of human monocytes with BCG prior to differentiation into DCs [21]. Treatment of PBMCs with peptides derived from the regions of difference (RD) antigens 12, 13 and 15 expressed by Mtb but not BCG led to Th2 cytokine production [22]. Moreover, the presence of regulatory T cells and Th2 cells may be lined to infection susceptibility to Mtb [23]. In fact, patients suffering from diabetes mellitus are more susceptible to tuberculosis development and this could be linked to a Th2-bias of their anti-Mtb responses [24]. We provide evidence for two novel Mtb antigens (30 kDa and 38-kDa) that induce Th2 responses *in vitro* in spite of their capacity to elicit only incomplete DC maturation.

## Conclusions

Here we showed that the 30-kDa and 38-kDa antigens as Mtb components induce only a partial DC maturation leading to a Th2 shift in T cell polarization assays. Since efficient immune protection from intracellular bacteria such as Mtb depends on IFN-γ dependent CD4^+^ Th1 and cytotoxic CD8^+^ T cell responses, the observed Th2 shift can be envisaged as an immune escape mechanism by Mtb. Thus, using the two 30-kDa and 38-kDa antigens as an Mtb vaccines may lead to a similar Th2 shift in vivo. Therefore these findings may have implications for vaccination strategies against Mtb [3] or for the pathological outcomes observed in Mtb infected individuals suffering also from other diseases such as diabetes [24]. Although Mtb interferes with several innate immune receptors to raise an immune response [25] it has developed several mechanisms of immune evasion strategies to prevent elimination [26]. Although infected individuals generate immune responses and most people can control the infection they never reach complete Mtb elimination and microbial sterility [27]. Thus, in most cases Mtb infections represent a fine balance between immune reaction and immune evasion. The latter mechanisms may also be envisaged to prevent immune pathology by too strong immune reactions. Together this reflects the co-evolution strategy of Mtb [28]. To develop effective vaccines without strong side effects this fine balance and all participating mechanisms have to be considered. A large part of the immune evasion by Mtb is based on the persistent or latent infection of macrophages and the TLR-2-dependent down-regulation of antigen presenting function by Mtb in these macrophages [29]. The emerging role of DCs as regulators of tuberculosis disease has been elucidated only during the last years by genome-wide transcriptome analyses [28]. It is well possible that also the induction of suboptimal maturation of DCs reflects another mechanism of Mtb to target antigen presenting cells. In our system we studied selective Mtb antigens and found that the two antigens 30-kDa and 38-kDa of Mtb may be involved in immune evasion at the level of DCs. The quality of DC manipulation by Mtb appears to differ from what is known from macrophages. While for macrophages the MHC-dependent antigen presenting function seems to dominate our data point to an intact antigen presentation as observed in the allo-MLR and SEB-priming, but a bias towards Th2 immunity. These *in vitro* data require now further validation *in vivo* but may indicate that the 30-kDa and 38-kDa antigens represent suboptimal Mtb vaccine candidates.

## Methods

### Dendritic cell generation from PBMC

Peripheral blood was depleted of thrombocytes and plasma by Trima Accel® apheresis system (CaridianBCT, #80300) and obtained from the Blood Bank of the University of Würzburg from healthy blood donors with consent of the Ethical Committee of the University of Wuerzburg. PBMC were isolated by centrifugation in Lymphocyte Separation Medium LSM 1077 (PAA Laboratories GmbH, Cölbe) and plated in sterile Tissue culture dishes 100 mm (Greiner No 664160) at a density of 5×10^7^ cells per dish in 10 ml culture medium: RPMI 1640 without L-Glutamine (PAA Laboratories GmbH, Cölbe) with 1% heat-inactivated human AB-Serum (PAA Laboratories GmbH, Cölbe), 2 mM L-Glutamin (PAA 200 mM) and Penicillin/Streptomycin (PAA 100x) and incubated at 37°C, 5% CO_2_ for 1 h. After 1 h the non-adherent-fraction (NAF) was removed and deep-frozen at −80°C for later use as T cell source for the allogeneic mixed lymphocyte reaction (MLR). Then the dishes were washed twice by gentle rinsing with PBS without Ca^2+^ or Mg^2+^. Afterwards 10 ml of fresh, warm culture medium was added on the dish with the adherent monocytes and incubated for overnight at 37°C, 5% CO_2_. At day one the culture medium was carefully removed that loosely adherent cells remained unaffected and new culture medium containing 800 U/ml GM-CSF (Leukine® Sargramostim, Bayer) and 250 U/ml IL-4 (Strathmann Biotec AG Hamburg) was added. Cytokines were added again at day 3 in 3 ml fresh medium (containing GM-CSF 800 U/ml and IL-4 250 U/ml) per dish and cultured until day 5.

### Mtb and other substances for DC maturation

H37Ra and H37Rv: CFPs of Mtb H37Rv and H37Ra were isolated by growing tubercle bacilli in Sauton’s synthetic medium as a stationary pellicle culture. Briefly, culture supernatants were centrifuged at 15,000 × g for 1 h, sterilized by filtration (0.22-μm pore size), and concentrated by ultrafiltration (Amicon Ultra-15 centrifugal filter unit with a 10-kDa-molecular- mass cutoff; Millipore). Polymyxin B columns were used to remove endotoxin in mycobacterial antigens after purification followed by sterile filtration. The 30-kDa antigen (Ag85 B complex) and 38-kDa antigens were purified from 6-week culture filtrates of Mtb H37Rv.

At day 5 the immature DC were harvested and distributed into 24-well-plates (Greiner) at a density of 5×10^5^ cells per well in 1 ml culture medium without cytokines. These DCs were stimulated with different mycobacterial antigens at 10 μg/ml, LPS (*E. coli* 0127:B8, Sigma) at 0.1 μg/ml or left unstimulated as a negative control. To determine the cytokine production of DCs, the supernatants of stimulated cells were collected and tested by ELISA kits for IL-6 (R&D Systems), IL-10 (R&D Systems), IL-12p40 and IL-12p70 (BD Biosciences).

### FACS analysis

DC were matured or left untreated at day 5 and phenotyped at day 7 with a panel of antibodies detecting the following surface markers: CD80 PE (mIgG1), CD83 APC (mIgG1), CD86 FITC (mIgG1) and HLA DR FITC (mIgG2a) (all BD Biosciences). For the staining 5×10^4^ cells were incubated with the antibodies and the respective isotype control was diluted 1:100 with buffer (PBS 0,1% BSA, 0,2% sodium azide, 0,5 M EDTA) at 4°C for 30 min. Then the cells were washed and analysed with an LSR II flow cytometer (BD) and alalyzed with FlowJo Cytometry Analysis Software (Tree Star Inc. Olten).

### Primary allogeneic MLR

At day 5 of DC culture NAF from allogeneic blood donors were prepared for allogeneic MLR and cultured over night. At day 6 the DCs matured with the stimuli indicated in the figure legend (see also above) were harvested and seeded into two 96-well-flatbottom-plates as triplicates at titrated amounts of DCs as indicated. Then 2× 10^5^ NAF from a different donor were added. After 3 days cultures were pulsed with 1 μCi/well [^3^H]-Thymidine (Hartmann Analytic, Braunschweig) for 16 h and then harvested onto glass fiber filter mats (PerkinElmer, USA), scintillation wax added and counted in a 1450 Microbeta Counter (Tomtec) to measure cell proliferation.

### Stimulation of secondary SEB-specific responses

At day 5 of DC culture the frozen autologous NAF were thawed and cultured over night. DCs were plated in two 96-well-flatbottom-plates as triplicates at titrated amounts. Responder cells were added at 2×10^5^ NAF from the same donor with 10 or 100 ng/ml *Staphylococcus aureus enterotoxin B* (SEB) (Sigma Aldrich). In parallel, for the secondary SEB stimulation, 4×10^4^ DCs were plated in 24-well-plates together with 1×10^6^ NAF cells per well in culture medium containing 10 ng/ml SEB. SEB showed a very mild maturation effect on DCs (not shown). Six days later T cell cultures were harvested, distributed into 96-well-plates at a density of 2×10^5^ cells per well and restimulated with 10 ng/ml PMA (Sigma Aldrich) and 500 ng/ml Ionomycin (Sigma Aldrich, *Streptomyces conglobatus*). After 3 days supernatants were collected for analysis of cytokine production of IL-10 (R&D Systems), IL-4 and IFN-γ (BD Biosciences) by ELISA kits.

### Statistics

Statistical significance was tested with the Prism® software (version 4.0a, GraphPad Inc.) using one-way ANOVA test with Bonferroni’s Multiple Comparison post test.

## Abbreviations

DC: Dendritic cells; Mtb: *Mycobacterium tuberculosis*; SEB: *Staphylococcus enterotoxin B*.

## Competing interests

The authors declare that they have no competing interests.

## Authors’ contributions

MH and ASB carried out the immunological studies. JSL prepared the Mtb products. ER and MBL drafted the manuscript. ER participated in the design of the study. MBL performed the statistical analysis. EKJ and MBL conceived of the study, and participated in its design and coordination and helped to draft the manuscript. All authors read and approved the final manuscript.
